# Caloric restriction protects against electrical kindling of the amygdala by inhibiting the mTOR signaling pathway

**DOI:** 10.3389/fncel.2015.00090

**Published:** 2015-03-11

**Authors:** Bryan V. Phillips-Farfán, María del Carmen Rubio Osornio, Verónica Custodio Ramírez, Carlos Paz Tres, Karla G. Carvajal Aguilera

**Affiliations:** ^1^Laboratorio de Nutrición Experimental, Instituto Nacional de PediatríaMéxico City, México; ^2^Laboratorio de Neurofisiología, Instituto Nacional de Neurología y NeurocirugíaMéxico City, México

**Keywords:** caloric restriction, epilepsy, mTOR signaling cascade, hippocampus, neocortex

## Abstract

Caloric restriction (CR) has been shown to possess antiepileptic properties; however its mechanism of action is poorly understood. CR might inhibit the activity of the mammalian or mechanistic target of rapamycin (mTOR) signaling cascade, which seems to participate crucially in the generation of epilepsy. Thus, we investigated the effect of CR on the mTOR pathway and whether CR modified epilepsy generation due to electrical amygdala kindling. The former was studied by analyzing the phosphorylation of adenosine monophosphate-activated protein kinase, protein kinase B and the ribosomal protein S6. The mTOR cascade is regulated by energy and by insulin levels, both of which may be changed by CR; thus we investigated if CR altered the levels of energy substrates in the blood or the level of insulin in plasma. Finally, we studied if CR modified the expression of genes that encode proteins participating in the mTOR pathway. CR increased the after-discharge threshold and tended to reduce the after-discharge duration, indicating an anti-convulsive action. CR diminished the phosphorylation of protein kinase B and ribosomal protein S6, suggesting an inhibition of the mTOR cascade. However, CR did not change glucose, β-hydroxybutyrate or insulin levels; thus the effects of CR were independent from them. Interestingly, CR also did not modify the expression of any investigated gene. The results suggest that the anti-epileptic effect of CR may be partly due to inhibition of the mTOR pathway.

## Introduction

At first glance neurodegenerative diseases and epilepsy may seem to have little in common. However, upon further research it is clear that they are surprisingly similar. As an example, abnormal neural activity -the hallmark of epileptic disorders- might cause the synaptic and cognitive deficits observed in neurodegenerative diseases (Sanchez et al., 2012). Another unexpected commonality between neurodegenerative diseases and epilepsy may be a defect of autophagy (Wong, 2013; Lipton and Sahin, 2014) due to dysfunction of the mammalian or mechanistic target of rapamycin (mTOR) signaling pathway (Wong, 2013; Lipton and Sahin, 2014).

The ketogenic diet is a beneficial treatment for epilepsy that is frequently used in the clinic (Kossoff and Wang, 2013; Wang and Lin, 2013). Similarly, caloric restriction (CR) is a diet that has anti-epileptic and anti-epileptogenic effects in different animal models (Greene et al., 2001; Bough et al., 2003). Their mechanism of action is poorly understood; however it has been reported that the ketogenic diet might inhibit the mTOR cascade (McDaniel et al., 2011). Excessive activation of this pathway has been observed in diverse models of genetic or acquired epilepsy, suggesting that aberrant function of this cascade plays a crucial role in epilepsy generation (Wong, 2013; Lipton and Sahin, 2014). CR may also inhibit the mTOR pathway in yeast, flies and nematodes (Kapahi et al., 2004; Kaeberlein et al., 2005; Walker et al., 2005).

The mTOR cascade regulates protein synthesis and cell growth, among other things (**Figure 4**; Wong, 2013; Lipton and Sahin, 2014). Insulin and growth factors result in protein kinase B (PKB or *Akt*) phosphorylation, which inhibits tuberin (tuberous sclerosis 2 or *TSC2*). In contrast, *TSC2* is activated by adenosine monophosphate-activated protein kinase (AMPK) in response to energy deficits. *TSC2*, together with hamartin (tuberous sclerosis 1 or *TSC1*), inhibits the activity a complex which includes the mTOR protein. This complex activates ribosomal protein S6 kinase (*S6K*), which phosphorylates ribosomal protein S6 (S6). These proteins only function when phosphorylated at specific residues. Therefore, the only way to study their activity with the western blot technique is to probe with antibodies that only can recognize the protein when phosphorylated at one or more of these residues.

CR may not hinder the mTOR cascade in all mammalian tissues (Sharma et al., 2012). We thus studied whether CR inhibits the mTOR pathway in the hippocampus and the adjacent temporal neocortex to clarify this issue. This was done by analyzing the phosphorylation of the α1/2 subunits of AMPK, PKB and S6 using the western blot technique. In addition, we also investigated if CR modified the expression of genes encoding for proteins participating in the mTOR cascade. This pathway is regulated by energy and insulin levels, which might be changed by CR; thus we also studied whether CR altered the blood levels of glucose or β-hydroxybutyrate (β-HB) or the plasma concentration of insulin. Finally, we investigated the anti-epileptogenic effects of CR using electrical kindling of the amygdala, which allows investigation of epilepsy generation. The hippocampus and the adjacent temporal neocortex were chosen because the amygdala projects to the hippocampus and the adjacent temporal neocortex presumably includes hippocampal projection areas. In opposition, the amygdala was not analyzed to minimize any possible influence of electrode placement within it.

Most prior studies used weanling animals, since the ketogenic diet might be more effective the younger the animal (Bough et al., 1999). Thus, we used young rats due to the precedent set by previous reports. Additionally, the susceptibility to epilepsy is very high in childhood (Kotsopoulos et al., 2002). CR was accomplished by food restriction without vitamin or mineral supplementation; therefore the rats were subjected to mild CR (15%) to avoid any concerns relating to under-nutrition, especially given their young age. This low degree of CR was also selected given prior studies (Greene et al., 2001; Raffo et al., 2008; Linard et al., 2010).

## Materials and methods

### Animal handling

20 male Wistar rats were purchased from Harlan Laboratories at postnatal day 21 and then subjected to a 4 h fast to insure that all animals started at a similar metabolic set point. Afterwards, the rats were weight-matched and assigned to four groups: (1) 5 fed *ad libitum* (control), (2) 5 allowed to feed *ad libitum* and subjected to the kindling procedure (kindled control), (3) 5 calorically restricted (experimental) and (4) 5 subjected to CR plus kindling (kindled experimental). CR was achieved by feeding rats with a normal diet (2018S, Harlan Laboratories) to 85% of the daily allowance based on the body weights (recorded every day) of rats allowed to feed *ad libitum* (Rogers, 1979). The animals were kept 30 days on their respective diets before stereotaxic surgery; each group was housed separately under standard conditions. Rat management was performed according to the principles of the guide for the care and use of laboratory animals and NIH publication 85-23, 1985.

### Stereotaxic surgery

A group of rats fed *ad libitum* and a group of animals subjected to CR were operated, while the other 2 groups served as control groups. After deep ketamine (40 mg/kg) anesthesia was achieved, bipolar electrodes were implanted within the left baso-lateral amygdala and right sensory cortex (stereotaxic coordinates: anterior 2.8, 6.7 mm; lateral 5, 2.5 mm; ventral 8.5, 9 mm; from bregma and the interaural line, respectively). The electrodes consisted of two twisted strands of stainless steel (0.005′diameter) coated with Teflon, except the tips which were separated by 0.02′. They were soldered to connectors and fastened to the skull with acrylic cement and screws, a screw served as an indifferent source of isoelectric reference. Electrode placement was verified by performing histological staining techniques after the animals were sacrificed.

### Electrical kindling of the amygdala

The after-discharge (AD) threshold, defined as the lowest electrical stimulus that elicited an amygdala AD lasting at least 5 s (from the end of the stimulus), was determined after the rats were allowed to recover from surgery for a period of at least 10 days. We employed an electronic circuit breaker device that switched to either the polygraph (Grass 78D, Grass Technologies) or the stimulator (Grass S88, Grass Technologies) connected to the electrode placed in the amygdala (1.8 cm long). This allowed us to stimulate and record through this electrode (Ferrer et al., 1978). The electrode located within the sensory cortex (1.5 cm long) served to verify propagation of epileptic activity (supplementary figure). The electrical signal was amplified 1000x and processed with a 16-bit sigma delta converter at a sampling frequency of 256 Hz. The recordings were monitored and saved for off-line analysis with Harmonie v.5.2 software (Stellate Systems).

After 2 min of polygraphic recording to establish the basal activity, the amygdala was stimulated for 1 s with rectangular pulses of 1 ms at 60 Hz (Paz et al., 1991). The first stimulation intensity was 5 V followed by subsequent increments of 1 V until elicitation of an AD (the intensity eliciting an AD lasting at least 5 s from the end of the stimulus was defined as the AD threshold). In the following daily trials, the threshold intensity was applied until at least 5 consecutive generalized convulsive (stage 5) seizures were obtained. Seizure severity was scored according to behavioral criteria; briefly: stage 1-mouth and facial movements, stage 2-head nodding, stage 3-forelimb clonus, stage 4-rearing, stage 5-rearing and falling (Racine, 1972). We measured the following kindling parameters: AD threshold and duration, latency to each Racine stage (number of stimulations/days required to reach each Racine stage), latency to reach criterion (number of stimulations/days needed to reach 5 consecutive generalized stage 5 convulsive seizures), number of stimulations/days where the rats showed each Racine stage and number of stimulations/days the animals had either focal convulsive seizures (stages 1–3) or generalized convulsive seizures (stages 4–5). The intracranial electroencephalographic activity of the amygdala and the sensory cortex was captured using a signal capture software (Galileo, Stellate Systems) and merged with digital video records (Diva, Stellate Systems). All the animals were sacrificed the day after kindled rats were last stimulated (for a total of 64 days subjected to CR, postnatal day 85).

### Blood and plasma measurements

Glucose and β-HB levels were measured with a digital monitor system (MediSense Optium Xceed, Abbott Laboratories) after a 4 h fast, before beginning the experiment and once it ended, using blood samples collected from the tail vein. Blood was also obtained after a 4 h fast at sacrifice and processed to obtain plasma, which was kept at −70°C. The plasma was used to determine the insulin concentration by an enzyme-linked immunosorbent assay (Alpco Diagnostics) according to standard protocols.

### Hippocampus and temporal neocortex samples

The brain was obtained rapidly and the hippocampus (Hp) plus adjacent temporal neocortex (Cx) were micro-dissected quickly, separated and cut into two portions. The samples were frozen with liquid nitrogen and stored at −70°C. One piece was homogenized at 4°C with a lysis buffer (pH 7) containing (in mM): 50 Tris, 150 NaCl, 1 EGTA, plus phosphatase and protease inhibitors. After a 30 min incubation at 4°C, the homogenate was centrifuged at 16,000 g for 10 min at 4°C and the supernatant was recovered. The concentration of total protein was determined using the Lowry method.

The other fragment of each brain area was homogenized at 4°C with Trizol to obtain total RNA. Briefly, the protocol included chloroform addition, centrifugation at 12,000 g for 15 min at 4°C, isopropanol addition, centrifugation at 12,000 g for 10 min at 4°C and addition of ethanol followed by centrifugation at 7500 g for 10 min at 4°C. The rest of the brain was fixed with 4% paraformaldehyde, cryo-preserved in a 30% sucrose solution, frozen, cut and processed by histological staining techniques to verify electrode placement.

### Western blotting

Fifty Microgram/Microliter of protein was separated in a 10% SDS-PAGE gel under denaturing conditions, transferred to PVDF membranes and probed with: phospho-AMPKα (Thr172), AMPKα, phospho-PKB (Thr308), PKB/*Akt*, phospho-S6 (Ser235/236) or S6 (all from Cell Signaling Technology). The membranes were revealed utilizing a chemiluminescence assay and the amount of protein was analyzed utilizing Quantity One software (Bio-Rad Laboratories). Actin, detected with an antibody (Sigma-Aldrich Corporation), was used as loading control.

### Quantitative real-time polymerase chain reaction

Complementary DNA was obtained from total RNA by retro-transcription utilizing the M-MLV reverse transcriptase enzyme and random hexamers (Applied Biosystems) using the 2400 Geneamp PCR system (Perkin Elmer Inc.). The analysis of relative gene expression levels was done by quantitative real-time polymerase chain reaction using TaqMan gene expression assays. The procedure was performed following the instructions provided by the supplier (Applied Biosystems). The analyzed genes were: the α1 and α2 catalytic subunits of *AMPK*, *mTOR*, tuberous sclerosis 1 (*TSC1*), *TSC2* and ribosomal protein S6 kinase (*S6K*, polypeptide 1). The level of these genes was normalized against the expression of the gene that encodes the eukaryotic *18S rRNA* (endogenous control) using the difference in cycle threshold method with the StepOne real-time PCR system (Applied Biosystems).

### Statistics

Unpaired student's *t*-tests were performed to evaluate differences between kindled animals and those not subjected to kindling (control vs. kindled control, experimental vs. kindled experimental). All the variables behaved similarly between these groups (Supplementary Data). Thus, control and kindled control data were pooled together (hereafter AL, *n* = 10) and the results for experimental and kindled experimental rats were combined (henceforth CR, *n* = 10) for all subsequent analysis. Dissimilarities in body weight were evaluated utilizing two-way analysis of variance tests for each pair of days followed by Holm-Sidak *post-hoc* tests. The differences between groups (AL vs. CR) were evaluated using unpaired student's *t*-tests, whereas dissimilarities among groups (before vs. after) were analyzed with paired student's *t*-tests. A *p* ≤ 0.05 was considered to be significantly different. Figure data were expressed as mean ± standard error.

## Results

### Bio-indexes

**Figure 1:**
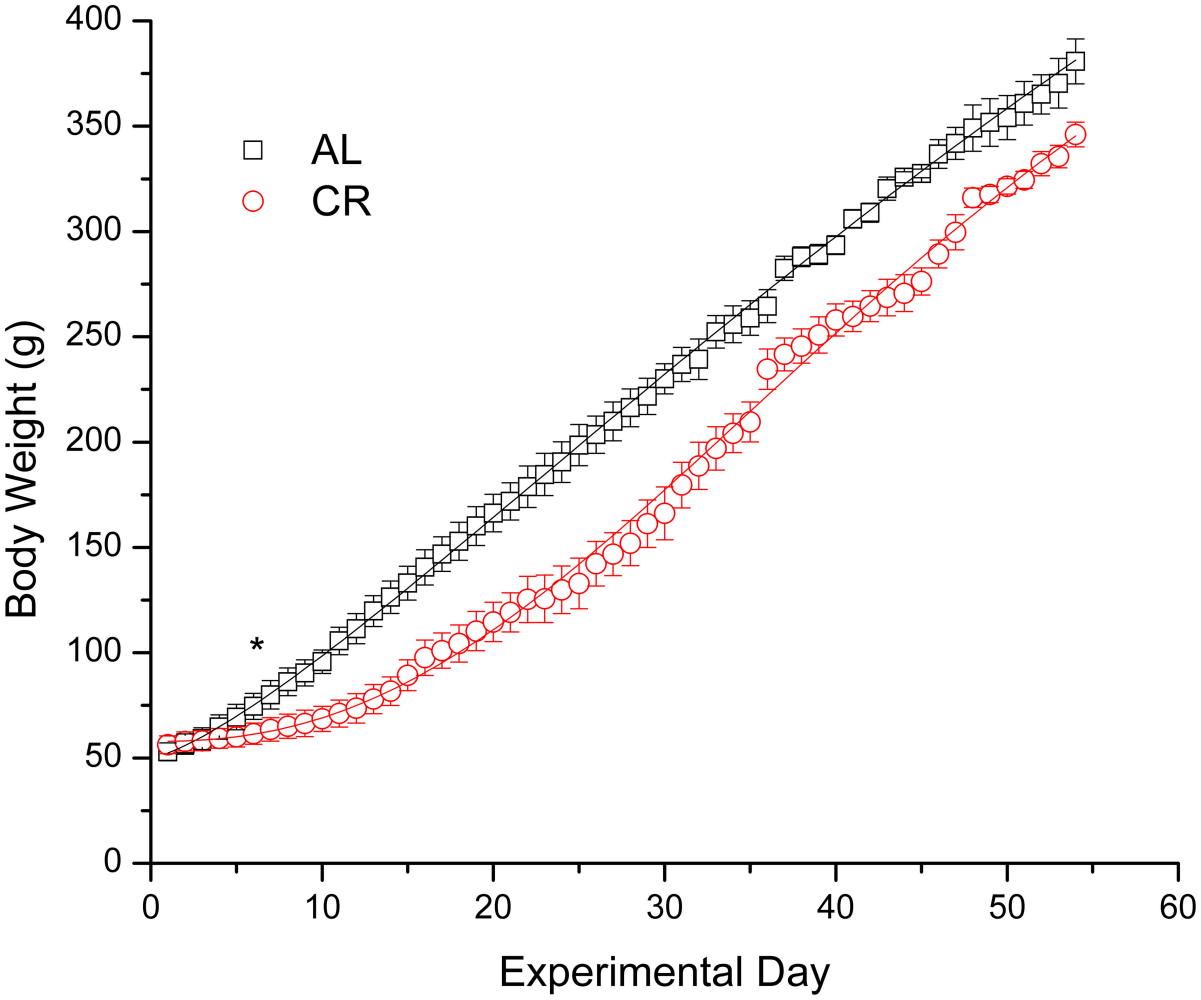
**Body weight gain in male rats fed *ad libitum* (AL) or subjected to 15% caloric restriction (CR) from postnatal day 21 (experimental day 1) to postnatal day 75 (day 54 of the procedure)**. The points were fit with logistic growth curves. The asterisk indicates the start of statistically significant differences (*p* < 0.05) among the groups (all other successive comparisons met this criterion).

**Figure 2:**
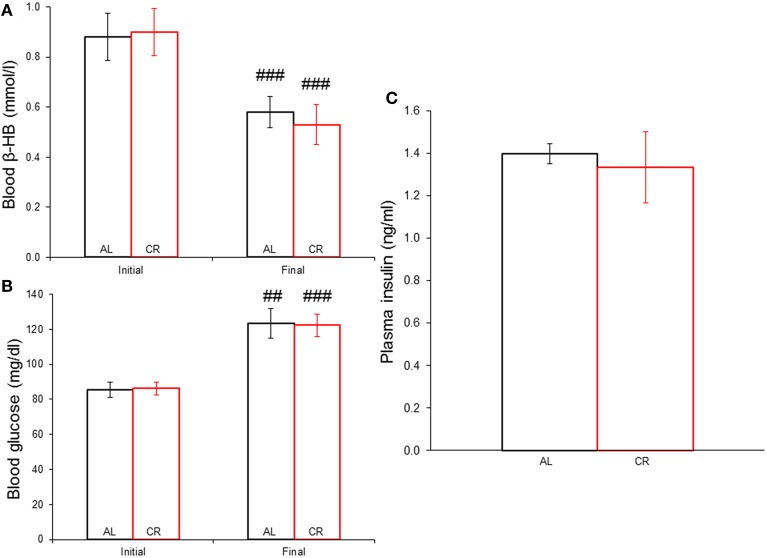
**Systemic concentration of energy substrates and the hormone insulin at the initial and final time points of the experiment in animals allowed food *ad libitum* (AL) or exposed to 15% caloric restriction (CR). (A)** Blood β-hydroxybutyrate (β-HB) levels (mmol/l). **(B)** Blood glucose concentrations (mg/dl). **(C)** Plasma insulin levels (ng/ml). ^##^*p* ≤ 0.01 and ^###^*p* ≤ 0.001, initial vs. final.

Body weights increased in both groups (Figure 1), but mild CR significantly reduced the weight gain of young animals [i.e., AL vs. CR in days 6 vs. 7: *F*_(1, 16)_ = 6.20, *p* < 0.05, *t* = 2.49; for all other comparisons see Supplementary Data]. On the other hand, fasting blood glucose and β-HB as well as plasma insulin (Figure 2) were similar between the groups [initial glucose: *t*_(1, 18)_ = −0.14, *p* = 0.89; final glucose: *t*_(1, 18)_ = 0.09, *p* = 0.93; initial β-HB: *t*_(1, 18)_ = −0.15, *p* = 0.88; final β-HB: *t*_(1, 18)_ = 0.50, *p* = 0.62; plasma insulin: *t*_(1, 18)_ = 0.371, *p* = 0.72]. Nonetheless, β-HB decreased whereas glucose increased significantly with time in both groups [initial vs. final; AL glucose: *t*_(1, 9)_ = −9.00, *p* ≤ 0.001; CR glucose: *t*_(1, 9)_ = −13.33, *p* ≤ 0.001; AL β-HB: *t*_(1, 9)_ = 4.03, *p* < 0.01 and CR β-HB: *t*_(1, 9)_ = 4.96, *p* ≤ 0.001].

### Electrical kindling of the amygdala

**Figure 3:**
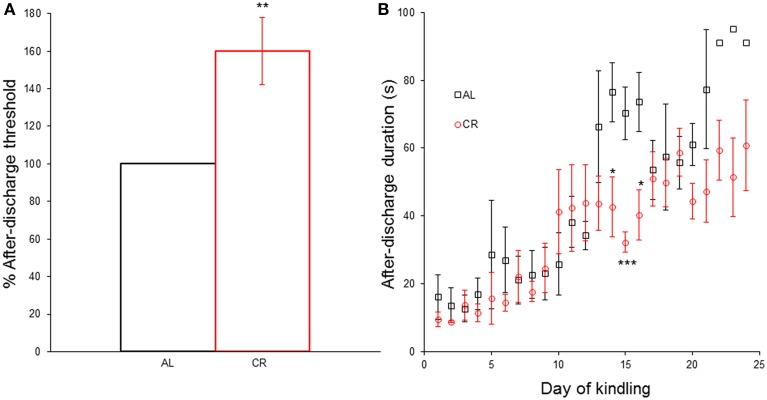
**Effect of caloric restriction (CR) upon electrical kindling of the amygdala. (A)** After-discharge threshold (%) determined the first day of the kindling procedure in rats fed *ad libitum* (AL) or subjected to 15% CR for at least 40 days. **(B)** After-discharge duration (in seconds) during kindling. ^*^p ≤ 0.05, ^**^p ≤ 0.01, ^***^p ≤ 0.001, AL vs. CR.

CR significantly increased [*t*_(1, 8)_ = −3.35, *p* = 0.01] the AD threshold (Figure 3A) and reduced the AD duration in days 14 [*t*_(1, 8)_ = 2.73, *p* < 0.05], 15 [*t*_(1, 8)_ = 4.63, *p* < 0.01] and 16 [*t*_(1, 8)_ = 2.92, *p* < 0.05] of the kindling procedure (Figure 3B). However, CR did not significantly alter the AD duration in any other day nor did it affect any other kindling parameter (latency to each Racine stage: AL 4.0 ± 1.0 vs. CR 3.8 ± 0.2 to stage 2, AL 8.7 ± 2.3 vs. CR 9.2 ± 1.1 to stage 3, AL 11.7 ± 1.2 vs. CR 12.8 ± 1.7 to stage 4, AL 13.7 ± 0.9 vs. CR 14.6 ± 2.0 to sage 5; latency to criterion: 17.7 ± 0.9 vs. 18.6 ± 2.0; days in each Racine stage: AL 3.0 ± 1.0 vs. CR 2.8 ± 0.2 in stage 1, AL 4.7 ± 1.5 vs. CR 5.4 ± 1.0 in stage 2, AL 3.0 ± 1.2 vs. CR 3.6 ± 1.4 in stage 3, AL 2.0 ± 1.0 vs. CR 1.8 ± 0.4 in stage 4, AL 9.3 ± 0.7 vs. CR 9.4 ± 0.7 in stage 5; days with focal convulsive seizures: AL 10.7 ± 1.2 vs. CR 11.8 ± 1.7; days rats had generalized convulsive seizures: AL 11.3 ± 0.3 vs. CR 11.2 ± 0.5; statistical comparisons in Supplementary Data).

### mTOR signaling pathway

**Figure 4:**
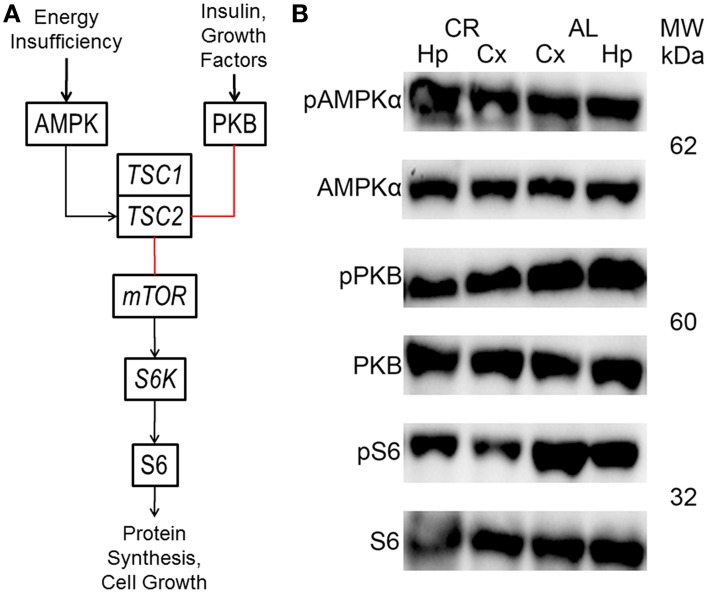
**(A)** Brief diagram of the mechanistic target of rapamycin (mTOR) pathway with analyzed proteins/genes (black arrows, activation; red lines, inhibition; AMPK, adenosine monophosphate-activated protein kinase; PKB, protein kinase B; *TSC1*, tuberous sclerosis 1; *TSC2*, tuberous sclerosis 2; *S6K*, ribosomal protein S6 kinase; S6, ribosomal protein S6). **(B)** Representative blots showing both phosphorylated (p) and total AMPK, PKB and S6 in the neocortex (Cx) and hippocampus (Hp) of rats fed *ad libitum* (AL) or subjected to 15% caloric restriction (CR). The molecular weight (MW) is shown in kilodaltons (kDa) to the right of each protein pair.

**Figure 5:**
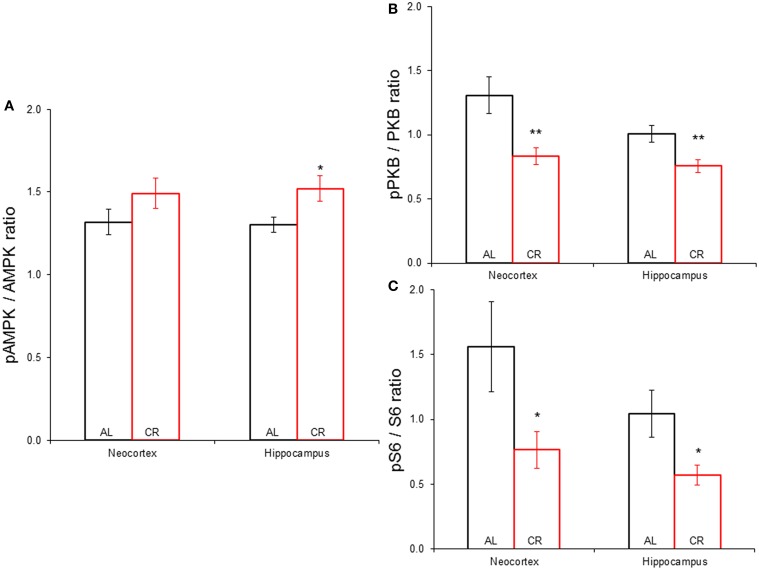
**Phosphorylation of proteins participating in the mechanistic target of rapamycin signaling pathway in the neocortex (Cx) and hippocampus (Hp) of animals allowed to feed *ad libitum* (AL) or subjected to a 15% caloric restriction (CR). (A)** Expression of phospho-AMPK against total AMPK. **(B)** Proportion of phospho-PKB vs. total PKB. **(C)** Phospho-S6 to total S6 ratio. ^*^p ≤ 0.05, ^**^p ≤ 0.01, AL vs. CR.

**Figure 6:**
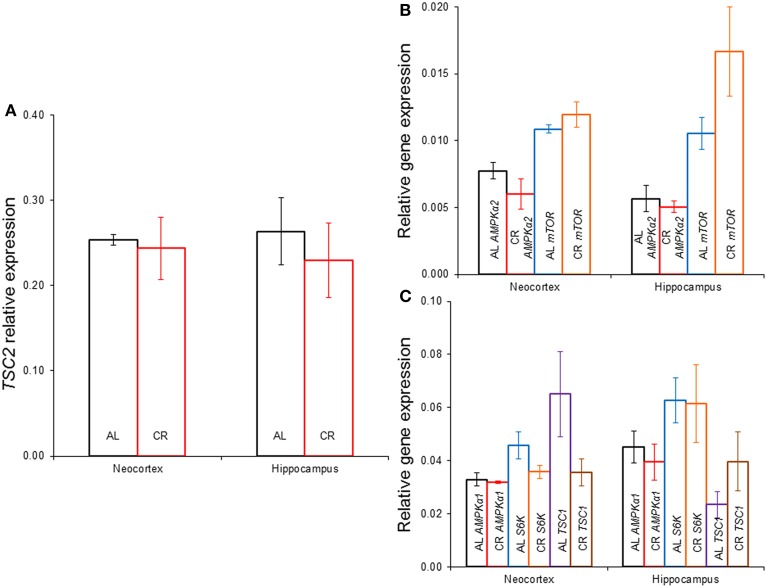
**Gene expression in the neocortex and hippocampus of rats fed *ad libitum* (AL) or exposed to 15% caloric restriction (CR). (A)** Comparison of tuberous sclerosis 2 (*TSC2*), **(B)** Semi-quantitative levels of the α*2* catalytic subunit of the adenosine monophosphate-activated protein kinase (*AMPK*) and the mechanistic target of rapamycin (*mTOR*) genes, **(C)** Relative level of the *α1* catalytic subunit of *AMPK*, ribosomal protein S6 kinase (*S6K*) and *TSC1* genes. There were no significant differences between the groups.

CR tended to augment AMPK phosphorylation (Figures 4, 5) in the Cx [*t*_(1, 18)_ = −1.47, *p* = 0.16] and significantly increased AMPK phosphorylation in the Hp [*t*_(1, 18)_ = −2.44, *p* < 0.05]. CR significantly reduced PKB/*Akt* and S6 phosphorylation in the Cx [PKB: *t*_(1, 18)_ = 3.03, *p* < 0.01 and S6: *t*_(1, 18)_ = 2.13, *p* < 0.05] and Hp [PKB: *t*_(1, 18)_ = 3.13, *p* < 0.01 and S6: *t*_(1, 18)_ = 2.39, *p* < 0.05], suggesting that CR inhibited the mTOR cascade. Finally, CR did not significantly change the expression level of the studied genes [Figure 6; *AMPKα1*: Cx: *t*_(1, 18)_ = 0.40, *p* = 0.70, Hp: *t*_(1, 18)_ = 0.62, *p* = 0.54; *AMPKα2*: Cx: *t*_(1, 18)_ = 1.37, *p* = 0.19, Hp: *t*_(1, 18)_ = 0.57, *p* = 0.58; *mTOR*: Cx: *t*_(1, 18)_ = −1.09, *p* = 0.29, Hp: *t*_(1, 18)_ = −1.71, *p* = 0.10; *S6K*: Cx: *t*_(1, 18)_ = 1.78, *p* = 0.09, Hp: *t*_(1, 18)_ = 0.08, *p* = 0.94; *TSC1*: Cx: *t*_(1, 18)_ = 1.74, *p* = 0.10, Hp: *t*_(1, 18)_ = −1.33, *p* = 0.20; *TSC2*: Cx: *t*_(1, 18)_ = 0.27, *p* = 0.79, Hp: *t*_(1, 18)_ = 0.58, *p* = 0.57].

## Discussion

### Body weight

Interestingly, mild CR significantly diminished weight gain of weanling rats; therefore our concerns with under-nutrition were well founded. This is not a universal finding given that some studies have shown a similar decrease in body weight (Greene et al., 2001; Cheng et al., 2003, 2004; Eagles et al., 2003), whereas other reports have not (Bough et al., 1999, 2003; Raffo et al., 2008). These contradictory findings have been mostly ignored, perhaps because little explanation can be offered as to the reasons for these discrepancies. The age at diet onset and length of time on it are the most likely factors that may account for the inconsistent results regarding the effect of CR on body weight. It is important to note that a reduction in body weight gain is consistent with an inhibition of the mTOR pathway. Even if a decrease in body weight was observed, CR showed anti-epileptic actions in all reports. Thus, it is unlikely the animals were malnourished given the adverse effects of malnutrition on epilepsy (Crepin et al., 2009).

### Biochemical parameters

Blood glucose or β-HB and plasma insulin were similar between the groups, again showing that CR did not result in under-nutrition. The normal developmental profile of increasing glucose and decreasing β-HB levels was observed (Edmond et al., 1985; Prins, 2012). CR has not been reported to affect blood β-HB levels in rats (Bough et al., 1999; Cheng et al., 2003, 2004; Eagles et al., 2003; Raffo et al., 2008; Linard et al., 2010), except for one report (Bough et al., 2003). On the other hand, Sprague-Dawley rats exposed to CR starting at weaning for 7 days had lower blood glucose compared to animals fed *ad libitum* (Cheng et al., 2003, 2004). In contrast, no significant alterations were seen in blood glucose levels of Wistar rats subjected to CR for 3 weeks starting at postnatal day 50 (Raffo et al., 2008; Linard et al., 2010). The most probable explanation is that the animals in the latter studies and ours adapted their metabolism to CR due to its duration, though other factors (species, age at diet onset) might account for the discordant findings. For example, CR significantly increased blood β-HB and decreased glucose levels in mice; suggesting a species difference (Greene et al., 2001; Mahoney et al., 2006). The finding that CR did not modify glucose, β-HB or insulin levels is very important because any effects of CR are thus independent from them.

### Electrical kindling of the amygdala

CR significantly increased the after-discharge threshold and diminished the after-discharge duration in a few days of the kindling procedure, but did not meaningfully alter any other kindling parameter. The number of stimulations to a generalized convulsive seizure (stage 5), known as the kindling rate, is a measure of the epileptogenic capability in this model. 15% CR did not alter the kindling rate in Wistar rats, thus CR was not anti-epileptogenic in these outbred animals. The anti-epileptogenic action of CR has been validated in mice and rats inbred for epileptic susceptibility (Greene et al., 2001; Azarbar et al., 2010), generating doubts whether CR really can be anti-epileptogenic in humans. However, a higher degree of CR likely has anti-epileptogenic effects in outbred animal models and humans. This is to be expected if a higher degree of CR has more prominent inhibitory effects on the mTOR signaling cascade. Alternatively, the anti-epileptogenic action of CR may require reductions in glucose or insulin and/or an increase in β-HB (Greene et al., 2001; Yamada, 2008).

Increases in the after-discharge threshold can be predictive of the anti-convulsive capacity of drugs in pharmacological tests (Azarbar et al., 2010). Similarly, since the after-discharge is an electrographic seizure, a reduction in its duration is an anti-convulsive effect (Azarbar et al., 2010). CR augmented the after-discharge threshold and reduced its duration, without affecting any other kindling variable (most importantly the kindling rate). This suggests an anti-convulsive profile rather than an anti-epileptogenic action; which is totally independent from changes in glucose, insulin or β-HB.

### mTOR signaling cascade

Mild CR significantly augmented AMPK phosphorylation in the hippocampus. Besides, it reduced PKB/*Akt* and S6 phosphorylation in the temporal neocortex and the hippocampus; suggesting that even mild CR inhibits the mTOR signaling pathway. Similarly, mice which were subjected to 30% CR starting at 3 months of age for 9 or 17 months showed reduced PKB/*Akt* and S6K phosphorylation levels in the hippocampus, suggesting that CR hindered this cascade (Yang et al., 2014). In contrast, 35–40% CR starting at 3–4 months of age for 6 months in Fischer 344 and Brown Norway F1 rats did not affect the phosphorylation level of several proteins that participate in this pathway in the cerebellum or neocortex (Sharma et al., 2012). These discordant findings are probably explained by the age at diet onset or its duration, although factors such as species, strain and brain region may have influenced the results.

The results suggest that CR inhibited the mTOR cascade in the hippocampus and adjacent temporal neocortex after the animals showed 5 consecutive generalized convulsive seizures (postnatal day 75). Thus, the status of the mTOR pathway in the amygdala when the after-discharge threshold (postnatal day 55) and duration (postnatal days 55–75) were determined is unknown. However, rats subjected to CR had been on this diet for at least 40 days before the kindling procedure started, thus CR probably likewise hindered the mTOR cascade in the amygdala. Furthermore, the effects of CR on the after-discharge threshold and duration were consistent with its known effects (Bough et al., 1999, 2003; Eagles et al., 2003; Azarbar et al., 2010) and consistent with regulation of neuron/network excitability by the mTOR cascade (Lasarge and Danzer, 2014; Lipton and Sahin, 2014).

Mild CR (15%) for 55 days starting on postnatal day 21 (this study) did not significantly change *AMPKα1*, *AMPKα2*, *mTOR*, *S6K*, *TSC1*, or *TSC2* expression in the hippocampus or temporal neocortex. In agreement, 20% CR for 6 months starting at 4 months of age did not significantly affect gene expression of proteins involved in the mTOR cascade in the hippocampus of Sprague-Dawley rats. However, 40% CR augmented the expression of genes involved in this pathway (Martin et al., 2008). These results show that the degree of CR is a very important factor determining the results. Similarly, 30–40% CR starting at 3–4 months of age for ≥6 months (Sharma et al., 2012; Yang et al., 2014) did not alter the level of mTOR, S6K or *TSC2* expression in the hippocampus, neocortex or cerebellum. But 30% CR for variable times (~4, 11, 18, or 23 months) starting at weaning reduced the expression of genes that participate in this cascade (such as S6K) in the hypothalamus of inbred mice (Wu et al., 2009). Given the similarity between two of these reports (Wu et al., 2009; Yang et al., 2014), the most likely explanation is that CR has differential impact depending on the age at diet onset or the brain region, as previously suggested (Zeier et al., 2011). Although CR and the ketogenic diet both inhibit the mTOR pathway (McDaniel et al., 2011), this is unlikely to be the only anti-epileptic mechanism of action of these treatments since they differ in important respects (Bough et al., 2000, 2003; Hartman et al., 2010). This is emphasized by the fact that the ketogenic diet has anti-epileptogenic effects (Jiang et al., 2012), whereas mild CR displayed a profile more consistent with an anti-convulsive action.

Our findings suggest using CR as a therapeutic intervention to inhibit the mTOR cascade in the human brain. The advantage of CR is that it can be combined with other treatments; this is generally done when using the ketogenic diet (Kossoff and Wang, 2013; Wang and Lin, 2013). Moreover, CR may be used for seizure-epilepsy prophylaxis; although more studies are needed to resolve current contradictory findings (Zeng et al., 2010; Macias et al., 2013). Unfortunately, even mild CR significantly reduced weight gain in weanling rats which may limit its use in children and adolescents due to concerns regarding their growth. In addition, despite the presumed inhibition of the mTOR pathway all the rats progressed to generalized convulsive seizures with the same kindling rate. This was probably due to the low degree of CR used; a higher amount of CR likely would result in anti-epileptogenic effects.

In summary, CR elevated the after-discharge threshold without changes in other kindling variables; suggesting an anti-convulsive action. CR increased AMPK phosphorylation in the hippocampus, while it decreased phosphorylation of PKB/*Akt* and S6 in the temporal neocortex and hippocampus; implying an inhibition of the mTOR signaling cascade. The effects of CR were likely independent from any changes in glucose, β-HB, insulin or gene expression (*AMPKα1*, *AMPKα2*, *mTOR*, *S6K*, *TSC1*, or *TSC2*) since none were modified by CR. Our main conclusion is that CR may inhibit the mTOR signaling cascade in the brain, which results in an anti-convulsive increase in convulsive seizure threshold that does not depend on changes in energy or insulin levels or gene expression of proteins that participate in this cascade. Thus, CR might be used as a therapeutic tool to inhibit the mTOR pathway to achieve anti-convulsive effects.

### Conflict of interest statement

The authors declare that the research was conducted in the absence of any commercial or financial relationships that could be construed as a potential conflict of interest.

## Supplementary material

Supplementary Figure **Fragment of an electrographic record, the space between each line represents one second**. The channels show the electrographic activity of the sensory cortex (SC) and the amygdala (A) before and after stimulating the amygdala. SA, stimulus artifact.

## Abbreviations

CR: caloric restriction
mTOR: mechanistic target of rapamycin
β-HB: β-hydroxybutyrate
AMPK: adenosine monophosphate-activated protein kinase
PKB/*Akt*: protein kinase B
S6: ribosomal protein S6
S6K: ribosomal protein S6 kinase
*TSC2*: tuberous sclerosis 2
AL: *ad libitum*
AD: after-discharge
Cx: neocortex
Hp: hippocampus.
