# Supplementary figures and images for: Caloric restriction protects against electrical kindling of the amygdala by inhibiting the mTOR signaling pathway
Source: Front Cell Neurosci. 2015 Mar 11;9:90. doi: 10.3389/fncel.2015.00090 (PMC4356078; doi:10.3389/fncel.2015.00090)

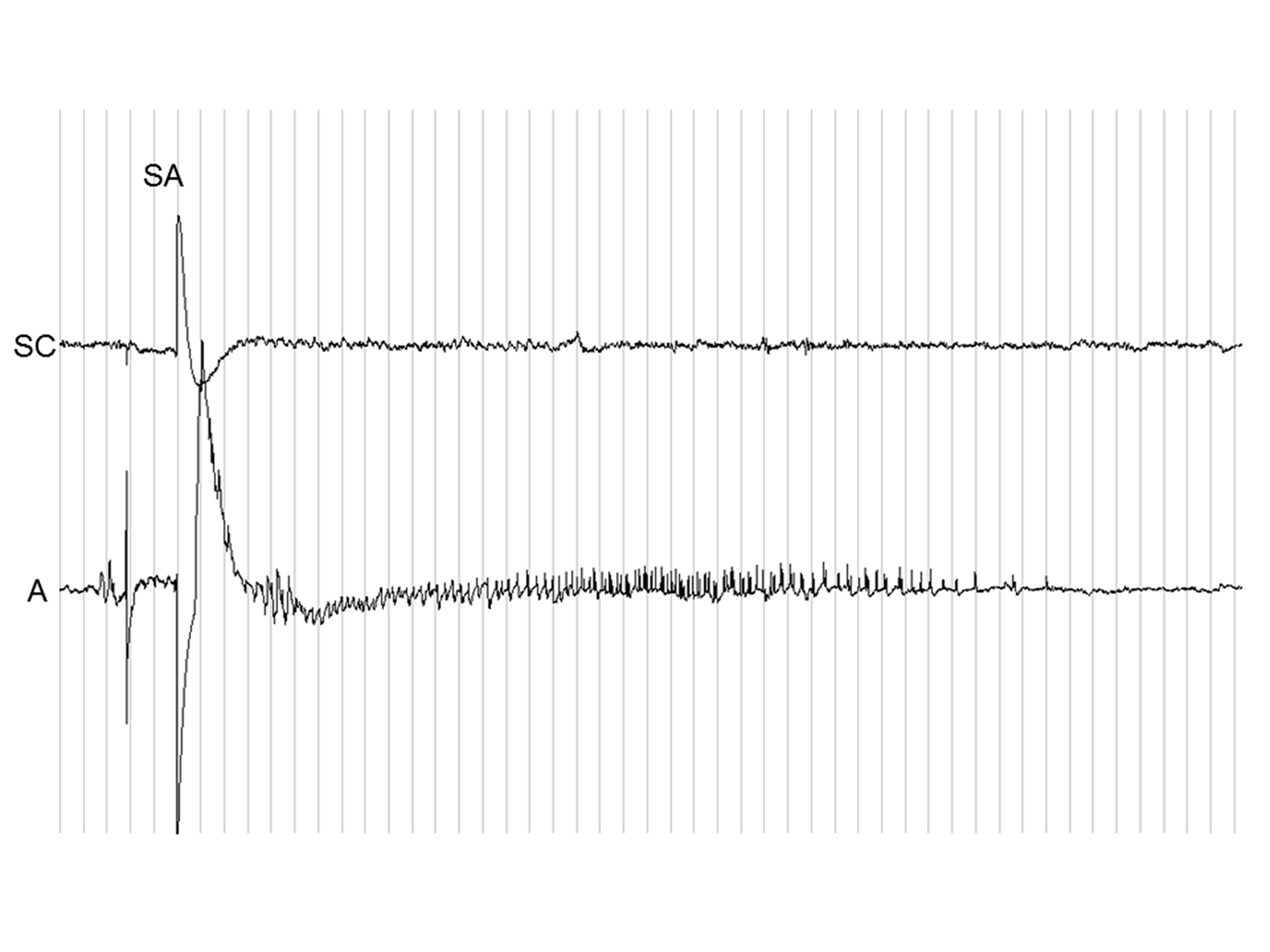

Supplement: Supplementary Figure — Fragment of an electrographic record, the space between each line represents one second. The channels show the electrographic activity of the sensory cortex (SC) and the amygdala (A) before and after stimulating the amygdala. SA, stimulus artifact. [file Image1.TIF]
