# Supplementary material for: Caloric restriction protects against electrical kindling of the amygdala by inhibiting the mTOR signaling pathway
Source: Front Cell Neurosci. 2015 Mar 11;9:90. doi: 10.3389/fncel.2015.00090 (PMC4356078; doi:10.3389/fncel.2015.00090)
Supplement: Supplementary file 2 [file DataSheet1.DOC]

**Supplementary data**

**A. Statistical comparisons between controls and kindled controls**

**Body Weights (Two-way ANOVA)**

Day 1 vs. 2: F_(1,16)_=0.421, p=0.526; Day 2 vs. 3: F_(1,16)_=0.936, p=0.348; Day 3 vs. 4: F_(1,16)_=1.001, p= 0.332; Day 4 vs. 5: F_(1,16)_=0.949, p=0.344; Day 5 vs. 6: F_(1,16)_=1.146, p=0.300; Day 6 vs. 7: F_(1,16)_=1.383, p=0.257; Day 7 vs.8: F_(1,16)_=1.516, p=0.236; Day 8 vs. 9: F_(1,16)_=1.386, p=0.256; Day 9 vs. 10: F_(1,16)_= 0.865, p=0.366; Day 10 vs. 11: F_(1,16)_=0.812, p=0.381; Day 11 vs. 12: F_(1,16)_=1.154, p=0.299; Day 12 vs. 13: F_(1,16)_=1.038, p=0.323; Day 13 vs. 14: F_(1,16)_=0.957, p=0.343; Day 14 vs. 15: F_(1,16)_=1.101, p=0.310; Day 15 vs. 16: F_(1,16)_=1.050, p=0.321; Day 16 vs. 17: F_(1,16)_=0.831, p=0.375; Day 17 vs. 18: F_(1,16)_=0.817, p=0.380; Day 18 vs. 19: F_(1,16)_=0.951, p=0.344; Day 19 vs. 20: F_(1,16)_=0.827, p=0.377; Day 20 vs. 21: F_(1,16)_=0.683, p=0.421; Day 21 vs. 22: F_(1,16)_=0.850, p=0.370; Day 22 vs. 23: F_(1,16)_=0.968, p=0.340; Day 23 vs. 24: F_(1,16)_=0.912, p=0.354; Day 24 vs. 25: F_(1,16)_=0.890, p=0.360; Day 25 vs. 26: F_(1,16)_=0.748, p= 0.400; Day 26 vs. 27: F_(1,16)_=0.772, p=0.393; Day 27 vs. 28: F_(1,16)_=0.921, p=0.351; Day 28 vs. 29: F_(1,16)_= 0.909, p=0.354; Day 29 vs. 30: F_(1,16)_=0.577, p=0.459; Day 30 vs. 31: F_(1,16)_=0.927, p=0.350; Day 31 vs. 32: F_(1,16)_=1.868, p=0.191; Day 32 vs. 33: F_(1,16)_=2.772, p=0.115; Day 33 vs. 34: F_(1,16)_=3.476, p=0.081; Day 34 vs. 35: F_(1,16)_=3.120, p=0.096; Day 35 vs. 36: F_(1,16)_=3.806, p=0.069; Day 36 vs. 37: F_(1,16)_=3.129, p=0.096; Day 37 vs. 38: F_(1,16)_=2.378, p=0.143; Day 38 vs. 39: F_(1,16)_=3.334, p=0.087; Day 39 vs. 40: F_(1,16)_=2.738, p=0.117; Day 40 vs. 41: F_(1,16)_=1.675, p=0.214; Day 41 vs. 42: F_(1,16)_=1.833, p=0.195; Day 42 vs. 43: F_(1,16)_=0.567, p=0.462; Day 43 vs. 44: F_(1,16)_=0.028, p=0.869; Day 44 vs. 45: F_(1,16)_=0.303, p= 0.590; Day 45 vs. 46: F_(1,16)_=0.001, p=0.977; Day 46 vs. 47: F_(1,16)_=0.271, p=0.610; Day 47 vs. 48: F_(1,16)_= 0.467, p=0.504; Day 48 vs. 49: F_(1,16)_=0.350, p=0.562; Day 49 vs. 50: F_(1,16)_=0.243, p=0.628; Day 50 vs. 51: F_(1,16)_=0.380, p=0.546; Day 51 vs. 52: F_(1,16)_=0.866, p=0.366; Day 52 vs. 53: F_(1,16)_=1.133, p=0.303; Day 53 vs. 54: F_(1,16)_=2.435, p=0.138

**Blood and plasma comparisons (Unpaired Student´s t test)**

Initial blood β-HB: 0.820±0.342 vs. 0.940±0.270, t_(1,8)_= -0.616, p=0.555; Final blood β-HB: 0.540±0.207 vs. 0.620±0.192, t_(1,8)_= -0.632, p=0.545; Initial blood glucose: 80.6±10.6 vs. 90.2±16.7, t_(1,8)_= -1.084, p= 0.310; Final blood glucose: 114±19.6 vs. 133±32.5, t_(1,8)_=-1.132, p=0.290; Plasma insulin: 1.36±0.131 vs. 1.44±0.164, t_(1,8)_= -0.889, p=0.400

**Western blot ratio comparisons (Unpaired Student´s t test)**

Cx pAMPK/AMPK: 1.27±0.211 vs. 1.37±0.296, t_(1,8)_= -0.624, p=0.550; Hp pAMPK/AMPK: 1.26±0.101 vs. 1.34±0.186, t_(1,8)_= -0.846, p=0.422; Cx pPKB/PKB: 1.27±0.444 vs. 1.35±0.494, t_(1,8)_=-0.283, p=0.784; Hp pPKB/PKB: 0.997±0.166 vs. 1.05±0.236, t_(1,8)_= -0.522, p=616; Cx pS6/S6: 1.42±1.05 vs. 1.71±1.26, t_(1,8)_= -0.403, p=0.698; Hp pS6 /S6: 0.989±0.612 vs. 1.09±0.609, t_(1,8)_= -0.269, p=0.795

**Quantitative real-time polymerase chain reaction, relative expression comparisons for each gene (Unpaired Student´s t test)**

Cx TSC2: 0.252±0.022 vs. 0.255±0.022, t_(1,8)_= -0.185, p=0.858; Hp TSC2: 0.251±0.130 vs. 0.276±0.129, t_(1,8)_= -0.308, p=0.766; Cx AMPKα2: 0.008±0.004 vs. 0.009±0.004, t_(1,8)_= -0.165, p=0.873; Hp AMPKα2: 0.006±0.004 vs. 0.007±0.004, t_(1,8)_= -0.197, p=0.849; Cx mTOR: 0.011±0.001 vs. 0.011±0.001, t_(1,8)_= -0.349, p=0.736; Hp mTOR: 0.010±0.002 vs. 0.011±0.002, t_(1,8)_= -0.512, p= 0.623; Cx AMPKα1: 0.031± 0.009 vs. 0.035±0.008, t_(1,8)_= -0.809, p=0.442; Hp AMPKα1: 0.044±0.018 vs. 0.047±0.022, t_(1,8)_= -0.222, p=0.830; Cx S6K: 0.046±0.016 vs. 0.047±0.016, t_(1,8)_= -0.129, p=0.900; Hp S6K: 0.057±0.025 vs. 0.068± 0.024, t_(1,8)_= -0.728, p=0.488; Cx TSC1: 0.057±0.050 vs. 0.073±0.057, t_(1,8)_= -0.483, p=0.642; Hp TSC1: 0.022±0.019 vs. 0.025±0.017, t_(1,8)_= -0.205, p= 0.842

**Quantitative real-time polymerase chain reaction, cycle threshold comparisons for 18S rRNA (Unpaired Student´s t test)**

Cx TSC2: 6.41±0.357 vs. 6.35±0.121, t_(1,8)_=0.430, p=0.678; Hp TSC2: 6.64±0.986 vs. 6.39±0.572, t_(1,8)_= 0.491, p=0.637; Cx AMPKα2: 7.07±0.744 vs. 7.28±0.853, t_(1,8)_= -0.408, p=0.694; Hp AMPKα2: 7.07± 1.07 vs. 6.98±0.577, t_(1,8)_=0.164, p=0.874; Cx mTOR: 7.51±0.205 vs. 7.51±0.193, t_(1,8)_= -0.027, p=0.979; Hp mTOR: 7.54±0.625 vs. 7.54±0.482, t_(1,8)_=0.006, p=0.996; Cx AMPKα1: 6.64±1.74 vs. 6.06±0.633, t_(1,8)_=0.701, p=0.503; Hp AMPKα1: 6.14±1.39 vs. 5.93±0.454, t_(1,8)_=0.329, p=0.751; Cx S6K: 7.49±0.485 vs. 7.53±0.472, t_(1,8)_= -0.119, p=0.908; Hp S6K: 7.81±0.988 vs. 8.05±1.06, t_(1,8)_= -0.368, p=0.723; Cx TSC1: 7.54±0.936 vs. 7.79±0.288, t_(1,8)_= -0.561, p=0.590; Hp TSC1: 7.46±1.17 vs. 8.02±0.781, t_(1,8)_= -0.904, p=0.393

**B. Statistical comparisons between experimental and kindled experimental groups**

**Body Weights (Two-way ANOVA)**

Day 1 vs. 2: F_(1,16)_=0.028, p=0.869; Day 2 vs. 3: F_(1,16)_=0.061, p=0.808; Day 3 vs. 4: F_(1,16)_=0.113, p= 0.741; Day 4 vs. 5: F_(1,16)_=0.218, p=0.647; Day 5 vs. 6: F_(1,16)_=0.316, p=0.582; Day 6 vs. 7: F_(1,16)_=0.580, p=0.458; Day 7 vs.8: F_(1,16)_=1.062, p=0.318; Day 8 vs. 9: F_(1,16)_=1.607, p=0.223; Day 9 vs. 10: F_(1,16)_= 1.721, p=0.208; Day 10 vs. 11: F_(1,16)_=1.708, p=0.210; Day 11 vs. 12: F_(1,16)_=1.844, p=0.193; Day 12 vs. 13: F_(1,16)_=1.887, p=0.189; Day 13 vs. 14: F_(1,16)_=1.735, p=0.206; Day 14 vs. 15: F_(1,16)_=1.347, p=0.263; Day 15 vs. 16: F_(1,16)_=0.910, p=0.354; Day 16 vs. 17: F_(1,16)_=0.710, p=0.412; Day 17 vs. 18: F_(1,16)_=0.839, p=0.373; Day 18 vs. 19: F_(1,16)_=0.901, p=0.357; Day 19 vs. 20: F_(1,16)_=0.851, p=0.370; Day 20 vs. 21: F_(1,16)_=0.870, p=0.365; Day 21 vs. 22: F_(1,16)_=0.889, p=0.360; Day 22 vs. 23: F_(1,16)_=1.212, p=0.287; Day 23 vs. 24: F_(1,16)_=1.603, p=0.224; Day 24 vs. 25: F_(1,16)_=2.059, p=0.171; Day 25 vs. 26: F_(1,16)_=2.184, p= 0.159; Day 26 vs. 27: F_(1,16)_=2.088, p=0.168; Day 27 vs. 28: F_(1,16)_=2.490, p=0.134; Day 28 vs. 29: F_(1,16)_= 2.511, p=0.133; Day 29 vs. 30: F_(1,16)_=2.537, p=0.131; Day 30 vs. 31: F_(1,16)_=2.620, p=0.125; Day 31 vs. 32: F_(1,16)_=1.898, p=0.187; Day 32 vs. 33: F_(1,16)_=1.777, p=0.201; Day 33 vs. 34: F_(1,16)_=3.165, p=0.094; Day 34 vs. 35: F_(1,16)_=4.362, p=0.053; Day 35 vs. 36: F_(1,16)_=3.036, p=0.101; Day 36 vs. 37: F_(1,16)_=2.547, p=0.130; Day 37 vs. 38: F_(1,16)_=3.282, p=0.089; Day 38 vs. 39: F_(1,16)_=3.134, p=0.096; Day 39 vs. 40: F_(1,16)_=1.677, p=0.214; Day 40 vs. 41: F_(1,16)_=0.829, p=0.376; Day 41 vs. 42: F_(1,16)_=1.082, p=0.314; Day 42 vs. 43: F_(1,16)_=1.603, p=0.224; Day 43 vs. 44: F_(1,16)_=1.992, p=0.177; Day 44 vs. 45: F_(1,16)_=2.259, p= 0.152; Day 45 vs. 46: F_(1,16)_=0.732, p=0.405; Day 46 vs. 47: F_(1,16)_=0.008, p=0.931; Day 47 vs. 48: F_(1,16)_= 0.033, p=0.858; Day 48 vs. 49: F_(1,16)_=0.720, p=0.409; Day 49 vs. 50: F_(1,16)_=1.612, p=0.222; Day 50 vs. 51: F_(1,16)_=2.065, p=0.170; Day 51 vs. 52: F_(1,16)_=2.387, p=0.142; Day 52 vs. 53: F_(1,16)_=2.612, p=0.126; Day 53 vs. 54: F_(1,16)_=3.186, p=0.093

**Blood and plasma comparisons (Unpaired Student´s t test)**

Initial blood β-HB: 0.840±0.305 vs. 0.960±305, t_(1,8)_=-0.622, p=0.551; Final blood β-HB: 0.480±0.192 vs. 0.580±0.311, t_(1,8)_=-0.611, p=0.558; Initial blood glucose: 82.8±11.5 vs. 89.6±12.3, t_(1,8)_=-0.902, p=0.393; Final blood glucose: 118±18.2 vs. 127±23.1, t_(1,8)_= -0.669, p=0.523; Plasma insulin: 1.30±0.573 vs. 1.37± 0.561, t_(1,8)_= -0.191, p=0.854

**Western blot ratio comparisons (Unpaired Student´s t test)**

Cx pAMPK/AMPK: 1.44±0.323 vs. 1.54±0.265, t_(1,8)_= -0.526, p=0.613; Hp pAMPK/AMPK: 1.47±0.215 vs. 1.56±0.283, t_(1,8)_= -0.580, p=0.578; Cx pPKB/PKB: 0.827±0.213 vs. 0.840±0.223, t_(1,8)_= -0.100, p= 0.922; Hp pPKB/PKB: 0.752±0.152 vs. 0.773±0.162, t_(1,8)_= -0.216, p=0.834; Cx pS6/S6: 0.736±0.461 vs. 0.776±0.476, t_(1,8)_= -0.135, p=0.896; Hp pS6/S6: 0.556±0.258 vs. 0.586±0.256, t_(1,8)_= -0.186, p=0.857

**Quantitative real-time polymerase chain reaction, relative expression comparisons for each gene (Unpaired Student´s t test)**

Cx TSC2: 0.232±0.119 vs. 0.255±0.121, t_(1,8)_= -0.298, p=0.774; Hp TSC2: 0.218±0.145 vs. 0.241±0.150, t_(1,8)_= -0.242, p=0.815; Cx AMPKα2: 0.006±0.004 vs. 0.007±0.004, t_(1,8)_= -0.162, p=0.875; Hp AMPKα2: 0.005±0.001 vs. 0.005±0.001, t_(1,8)_= -0.220, p=0.831; Cx mTOR: 0.012±0.003 vs. 0.013±0.003, t_(1,8)_= -0.240, p=0.816; Hp mTOR: 0.015±0.009 vs. 0.018±0.011, t_(1,8)_= -0.441, p= 0.671; Cx AMPKα1: 0.032± 0.001 vs. 0.032±0.002, t_(1,8)_= -0.186, p=0.857; Hp AMPKα1: 0.035±0.023 vs. 0.043±0.022, t_(1,8)_= -0.523, p=0.615; Cx S6K: 0.036±0.008 vs. 0.036±0.008, t_(1,8)_= -0.096, p=0.926; Hp S6K: 0.055±0.045 vs. 0.068± 0.053, t_(1,8)_= -0.414, p=0.690; Cx TSC1: 0.035±0.018 vs. 0.037±0.018, t_(1,8)_= -0.176, p=0.865; Hp TSC1: 0.036±0.038 vs. 0.045±0.038, t_(1,8)_= -0.373, p= 0.719

**Quantitative real-time polymerase chain reaction, cycle threshold comparisons for 18S rRNA (Unpaired Student´s t test)**

Cx TSC2: 6.05±0.669 vs. 6.00±0.444, t_(1,8)_=0.132, p=0.898; Hp TSC2: 5.83±0.325 vs. 5.84±0.308, t_(1,8)_= -0.018, p=0.986; Cx AMPKα2: 6.98±0.753 vs. 7.13±0.444, t_(1,8)_= -0.378, p=0.715; Hp AMPKα2: 6.80± 0.712 vs. 7.22±0.679, t_(1,8)_=-0.967, p=0.362; Cx mTOR: 7.21±0.288 vs. 7.19±0.214, t_(1,8)_=0.132, p=0.898; Hp mTOR: 7.12±0.251 vs. 7.35±0.571, t_(1,8)_= -0.845, p=0.423; Cx AMPKα1: 6.54±1.62 vs. 6.19±1.16, t_(1,8)_=0.390, p=0.706; Hp AMPKα1: 5.93±0.918 vs. 6.54±1.29, t_(1,8)_= -0.870, p=0.410; Cx S6K: 7.55± 0.691 vs. 7.99±0.741, t_(1,8)_= -0.963, p=0.364; Hp S6K: 7.18±0.761 vs. 7.99±1.61, t_(1,8)_= -1.024, p=0.336; Cx TSC1: 7.55±0.69 vs. 8.80±1.32, t_(1,8)_= -1.86, p=0.100; Hp TSC1: 6.89±1.09 vs. 7.36±0.954, t_(1,8)_= -0.717, p=0.494

**C. Comparisons between animals fed ad libitum and rats subjected to caloric restriction**

**Body weight (Two-way ANOVA, followed by Holm-Sidak post-hoc test)**

Day 1 vs. 2: F_(1,16)_=0.259, p=0.614; Day 2 vs. 3: F_(1,16)_=0.0005, p=0.983; Day 3 vs. 4: F_(1,16)_=0.397, p= 0.533; Day 4 vs. 5: F_(1,16)_=1.918, p=0.175; Day 5 vs. 6: F_(1,16)_=4.059, p=0.051; Day 6 vs. 7: F_(1,16)_=6.196, p=0.018, t=2.489; Day 7 vs.8: F_(1,16)_=9.227, p=0.004, t=3.038; Day 8 vs. 9: F_(1,16)_=13.18, p<0.001, t= 3.630; Day 9 vs. 10: F_(1,16)_=18.06, p<0.001, t=4.249; Day 10 vs. 11: F_(1,16)_=25.12, p<0.001, t=5.012, Day 11 vs. 12: F_(1,16)_=28.35, p<0.001, t=5.324; Day 12 vs. 13: F_(1,16)_=31.47, p<0.001, t=5.609; Day 13 vs. 14: F_(1,16)_=35.98, p<0.001, t=5.998; Day 14 vs. 15: F_(1,16)_=34.90, p<0.001, t=5.908; Day 15 vs. 16: F_(1,16)_= 29.07, p<0.001, t=5.391; Day 16 vs. 17: F_(1,16)_=28.31, p<0.001, t=5.321; Day 17 vs. 18: F_(1,16)_=30.11, p< 0.001, t=5.487; Day 18 vs. 19: F_(1,16)_=29.25, p<0.001, t=5.409; Day 19 vs. 20: F_(1,16)_=30.48, p<0.001, t= 5.521; Day 20 vs. 21: F_(1,16)_=33.09, p<0.001, t=5.753; Day 21 vs. 22: F_(1,16)_=29.68, p<0.001, t=5.448; Day 22 vs. 23: F_(1,16)_=28.48, p<0.001, t=5.337; Day 23 vs. 24: F_(1,16)_=31.86, p<0.001, t=5.644; Day 24 vs. 25: F_(1,16)_=34.55, p<0.001, t=5.878; Day 25 vs. 26: F_(1,16)_=37.48, p<0.001, t=6.122; Day 26 vs. 27: F_(1,16)_= 40.86, p<0.001, t=6.392; Day 27 vs. 28: F_(1,16)_=42.13, p<0.001, t=6.490; Day 28 vs. 29: F_(1,16)_=39.24, p< 0.001, t=6.264; Day 29 vs. 30: F_(1,16)_=37.85, p<0.001, t=6.153; Day 30 vs. 31: F_(1,16)_=37.49, p<0.001, t= 6.123; Day 31 vs. 32: F_(1,16)_=29.05, p<0.001, t=5.390; Day 32 vs. 33: F_(1,16)_=29.33, p<0.001, t=5.416; Day 33 vs. 34: F_(1,16)_=35.10, p<0.001, t=5.924; Day 34 vs. 35: F_(1,16)_=32.11, p<0.001, t=5.666; Day 35 vs. 36: F_(1,16)_=20.14, p<0.001, t=4.488; Day 36 vs. 37: F_(1,16)_=20.17, p<0.001, t=4.491; Day 37 vs. 38: F_(1,16)_= 38.18, p<0.001, t=6.179; Day 38 vs. 39: F_(1,16)_=35.12, p<0.001, t=5.926; Day 39 vs. 40: F_(1,16)_=31.15, p< 0.001, t=5.581; Day 40 vs. 41: F_(1,16)_=44.99, p<0.001, t=6.708; Day 41 vs. 42: F_(1,16)_=56.40, p<0.001, t= 7.510; Day 42 vs. 43: F_(1,16)_=51.23, p<0.001, t=7.157; Day 43 vs. 44: F_(1,16)_=60.45, p<0.001, t=7.775; Day 44 vs. 45: F_(1,16)_=87.74, p<0.001, t=9.367; Day 45 vs. 46: F_(1,16)_=72.05, p<0.001, t=8.488; Day 46 vs. 47: F_(1,16)_=37.13, p<0.001, t=6.093; Day 47 vs. 48: F_(1,16)_=21.05, p<0.001, t=4.588; Day 48 vs. 49: F_(1,16)_= 15.93, p<0.001, t=3.992; Day 49 vs. 50: F_(1,16)_=16.77, p<0.001, t=4.095; Day 50 vs. 51: F_(1,16)_=19.24, p< 0.001, t=4.387; Day 51 vs. 52: F_(1,16)_=19.71, p<0.001, t=4.439; Day 52 vs. 53: F_(1,16)_=15.91, p<0.001, t= 3.989; Day 53 vs. 54: F_(1,16)_=15.27, p<0.001, t=3.907

**Quantitative real-time polymerase chain reaction, cycle threshold comparisons for 18S rRNA (Unpaired Student´s t test)**

Cx TSC2: 7.64±0.691 vs. 8.17±1.12, t_(1,8)_= -1.279, p=0.217; Hp TSC2: 7.74±0.942 vs. 7.13±0.952, t_(1,8)_= 1.45, p=0.164; Cx AMPKα2: 7.17±0.749 vs. 7.04±0.596, t_(1,8)_=0.438, p=0.667; Hp AMPKα2: 7.03±0.835 vs. 7.01±0.683, t_(1,8)_=0.061, p=0.952; Cx mTOR: 7.51±0.375 vs. 7.20±0.485, t_(1,8)_=1.56, p=0.136; Hp mTOR: 7.54±0.532 vs. 7.24±0.427, t_(1,8)_=1.40, p=0.178; Cx AMPKα1: 6.39±1.32 vs. 6.39±1.34, t_(1,8)_= 0.001, p=0.999; Hp AMPKα1: 6.05±1.02 vs. 6.24±1.09, t_(1,8)_= -0.398, p=0.695; Cx S6K: 7.51± 0.428 vs. 7.77±0.684, t_(1,8)_= -1.01, p=0.328; Hp S6K: 7.93±0.924 vs. 7.58±1.21, t_(1,8)_=0.713, p=0.485; Cx TSC1: 7.64±0.691 vs. 8.17±1.12, t_(1,8)_= -1.28, p=0.217; Hp TSC1: 7.74±0.942 vs. 7.13±0.952, t_(1,8)_=1.451, p= 0.164

**After-discharge duration (Unpaired Student´s t test)**

Day 1: t_(1,8)_=0.966, p=0.362; Day 2: t_(1,8)_=0.955, p=0.368; Day 3: t_(1,8)_=-0.178, p=0.863; Day 4: t_(1,8)_= 1.073, p=0.315; Day 5: t_(1,8)_=0.731, p=0.485; Day 6: t_(1,8)_=1.254, p=0.245; Day 7: t_(1,8)_=-0.109, p=0.916; Day 8: t_(1,8)_=0.661, p=0.527; Day 9: t_(1,8)_=-0.143, p=0.890; Day 10: t_(1,8)_=-0.997, p=0.348; Day 11: t_(1,8)_= -0.279, p=0.788; Day 12: t_(1,8)_=-0.797, p=0.448; Day 13: t_(1,8)_=1.226, p=0.255; Day 14: t_(1,8)_=2.728, p= 0.026; Day 15: t_(1,8)_=4.632, p=0.002; Day 16: t_(1,8)_=2.921, p=0.019; Day 17: t_(1,8)_=0.221, p=0.831; Day 18: t_(1,8)_=0.438, p=0.673; Day 19: t_(1,8)_=-0.287, p=0.781; Day 20: t_(1,5)_=2.053, p=0.095; Day 21: t_(1,5)_=1.648, p=0.160

**Number of stimulations required (latency) to reach each Racine stage (Unpaired Student´s t test)**

To stage 2: 4.00±2.24 vs. 3.80±0.447, t_(1,8)_=0.196, p=0.0.849; To stage 3: 8.67±5.22 vs. 9.20±2.39, t_(1,8)_= -0.208, p=0.841; To stage 4: 11.7±2.69 vs. 12.8±3.70, t_(1,8)_=-0.554, p=0.595; To stage 5 (kindling rate): 13.7±1.97 vs. 14.6±4.39, t_(1,8)_=-0.433, p=0.0676

**Number of stimulations required to reach 5 consecutive generalized (stage 5) convulsive seizures (latency to reach criterion)**

17.7±1.97 vs. 18.6±4.39, t_(1,8)_=-0.433, p=0.676

**Number of stimulations that rats were in each Racine stage (Unpaired Student´s t test)**

In stage 1: 3.00±2.24 vs. 2.80±0.447, t_(1,8)_=0.196, p=0.0.849; In stage 2: 4.67±3.25 vs. 5.40±2.30, t_(1,8)_= -0.412, p=0.691; In stage 3: 3.00±2.58 vs. 3.60±3.21, t_(1,8)_=-0.326, p=0.753; In stage 4: 2.00±2.24 vs. 1.80 ±0.837, t_(1,8)_=0.187, p=0.856; In stage 5: 9.33±1.49 vs. 9.40±1.52, t_(1,8)_=-0.070, p=0.946

**Number of stimulations where rats had focal or generalized convulsive seizures (Unpaired Student´s t test)**

In Racine stages 1-3: 10.7±2.69 vs. 11.8±3.70, t_(1,8)_= -0.554, p=0.595; In Racine stages 4-5: 11.3±0.745 vs. 11.2±1.10, t_(1,8)_=0.225, p=0.828
